# ACKNOWLEDGEMENTS TO REVIEWERS

**DOI:** 10.2174/1573403X2006240828124549

**Published:** 2024-08-28

   Bentham Science Publishers would like to thank and appreciate the co-operation from all reviewers for their constructive comments and feedback on the manuscripts submitted to **“Current Cardiology Reviews”**. Their efforts have contributed greatly to the high quality and continuous growth of the journal. Given below is the list of reviewers who reviewed articles for the Journal during 2024:

Ahmed S. Abuzaid (USA)

Yuichiro Adachi (USA)

Alexander A. Aksenov (USA)

Antonio F. Amico (Italy)

Zhao An (China)

Ryuichiro Anan (Japan)

Ozlem Bilen (USA)

Ugur Canpolat (Turkey)

Jun Cheng (China)

Alberto Cresti (Italy)

Yan Dai (China)

Soghra Farzipour (Iran)

Junying Gao (China)

Masaki Hamamoto (Japan)

Lei Hou (China)

Mohammad H. Javanbakht (Iran)

Kouji Kajinami (Japan)

Shahzad Khan (Saudi Arabia)

Safir U. Khan (China)

Habib Khan (Canada)

James D. Kingsley (USA)

Kaan Kirali (Turkey)

Melvin E. Klegerman (USA)

Gerhard Kostner (Austria)

Richard Kovacs (USA)

Martin Landsberger (Germany)

Seung W. Lee (Korea, South)

Charles A. Lefkowitz (Canada)

Shaoxiong Li (China)

Chao Liu (China)

Hailei Liu (China)

Stella M. Macin (Argentina)

Nani Maharani (Indonesia)

Leonid N. Maslov (Russian Federation)

Farzad Masoudkabir (Iran)

Sidharth Mehan (India)

Maryam Mehrpooya (Iran)

Yasushi Mukai (Japan)

Thach Nguyen (USA)

Steve R. N. Noumegni (France)

Masaaki Okutsu (Japan)

Beatrice Paradiso (Italy)

Weihua Qiao (China)

Yanwen Qin (China)

Hugo R. Ramos (Argentina)

Umut Safer (Turkey)

Gaetano Santulli (USA)

Ferena Sayar (Iran)

Finney D. Shadrach (India)

Hongli Shan (China)

Changsheng Sheng (China)

Andrea Sonaglioni (Italy)

Punniyakoti V. Thanikachalam (India)

Joho Tokumine (Japan)

Munesh Tomar (India)

Bulent Turan (Turkey)

Huseyin Uyarel (Turkey)

Tao Wang (China)

Guohua Wang (China)

Mistiaen Wilhelm (Belgium)

Juefei Wu (China)

Chennian Xu (China)

Xianxiang Xu (China)

Farah Yasmin (USA)

Naufal S. Zagidullin (Russian Federation)

Li Zhang (China)

Zhan Zhong-Qun (China)

Weizhong Zhu (China)

